# Quality of life after necrotizing enterocolitis: an integrative review

**DOI:** 10.1590/1984-0462/2024/42/2023188

**Published:** 2024-04-29

**Authors:** Breno Oliveira Marques, Ana Beatriz Ferreira Gusmão, Luana Leal Gonzaga, Gabriela Garcia de Carvalho Laguna, Níkolas Brayan da Silva Bragas, Camila da Palma Maltez Monção, Natália Oliveira e Silva

**Affiliations:** a Universidade Federal da Bahia, Vitória da Conquista, BA, Brasil.

**Keywords:** Necrotizing enterocolitis, Quality of life, Infant, newborn, Enterocolite necrosante, Qualidade de vida, Recém-nascidos

## Abstract

**Objective:**

To describe the long-term health outcomes of neonates affected by necrotizing enterocolitis (NEC) and its implications for quality of life.

**Data source:**

This is an integrative review, conducted by searching the literature in the following databases: Virtual Health Library (BVS), Latin American and Caribbean Health Sciences Literature (LILACS), Medical Literature Analysis and Retrieval System Online (MEDLINE), and PubMed, using Health Sciences Descriptors (DeCS): “necrotizing enterocolitis,” “quality of life,” and “prognosis” combined with the Boolean operators AND and OR: “quality of life” OR “prognosis.” Inclusion criteria were: publication period between 2012 and 2022.

**Data synthesis:**

A total of 1,010 studies were located, of which ten were selected to comprise the bibliographic sample of this review. Children with NEC are prone to exhibit cognitive neurological impairment, especially those who undergo surgical procedures due to more severe conditions. Motor development was considered below average when compared to healthy children, with more noticeable delays in fine and gross motor function development. The search for the relationship between NEC and quality of life revealed that this condition has a negative impact on the well-being of affected individuals.

**Conclusions:**

NEC has proven to be a serious condition contributing to high rates of morbidity and mortality in newborns, potentially leading to a reduction in the quality of life of affected patients.

## RESUMO

**Objetivo:**

Descrever os desfechos em longo prazo na saúde de neonatos acometidos com enterocolite necrosante (ECN) e suas repercussões na qualidade de vida.

**Fontes de dados:**

Trata-se de uma revisão integrativa, realizada com base no levantamento da bibliografia nas bases de dados: Biblioteca Virtual em Saúde (BVS), Literatura Latino-Americana e do Caribe em Ciências da Saúde (Lilacs), Medical Literature Analysis and Retrieval System Online (Medline) e PubMed por meio dos Descritores em Ciências da Saúde (DeCS): “necrotizing enterocolitis”, “quality of life” e “prognosis” combinados com os operadores booleanos AND e OR: “quality of life” OR “prognosis”. Os critérios de inclusão foram: período de publicação entre 2012 e 2022;

**Síntese dos dados:**

Foram localizados 1.010 estudos, dos quais dez foram selecionados para compor a amostra bibliográfica desta revisão. As crianças com ECN são propensas a apresentar comprometimento neurológico cognitivo, principalmente aquelas submetidas a procedimentos cirúrgicos em razão de quadros mais graves. O desenvolvimento motor foi considerado abaixo da média se comparado ao de crianças saudáveis, com atraso mais perceptível no desenvolvimento de funções motoras finas e grossas. A busca pela relação entre enterocolite necrosante e qualidade vida trouxe como resultado que essa condição tem impacto negativo sobre o bem-estar dos indivíduos acometidos.

**Conclusões:**

A enterocolite necrosante mostrou-se como uma condição grave que contribui para altas taxas de morbimortalidade em recém-nascidos, sendo uma potencial causa para a redução da qualidade de vida dos pacientes acometidos.

## INTRODUCTION

Necrotizing enterocolitis (NEC) is an acquired inflammatory disease that predominantly affects the intestines of newborns and ranks among the most common and devastating conditions in preterm neonates, with an estimated mortality rate of 20 to 30%.^1^ This disease is the leading cause of surgical emergency in newborns and can manifest insidiously or suddenly, with approximately 90% of cases developing after the initiation of feeding.^2^

Affected neonates requiring surgical procedures are often associated with longer hospitalization periods, averaging about two months longer than those who do not require surgery.^3^ This extended neonatal stay in these units poses risks and consequences for the well-being of the child.

Key factors associated with NEC include immaturity of gastrointestinal motility and absorption, immune defense, and circulatory regulation.^4^ Genetic predispositions, atypical microbiota colonization, and high immunoreactivity of the intestinal mucosa also contribute to this complex condition.

Regarding disease complications, a noteworthy situation is the need for intestinal resection surgery, which is the primary cause of short bowel syndrome (SBS), significantly compromising nutrient absorption capacity.^5^

The significant events of this disease occur during infancy, a critical period for the development of the brain, formation of eating habits, establishment of social relationships, and acquisition of physical skills, making it a crucial time for future health and well-being.^6^ Necrotizing enterocolitis can have a lasting impact on the lives of affected children, resulting in a direct negative impact on their physical, psychological, and social aspects of quality of life. These consequences were elucidated by a study aimed at understanding how NEC affects survivors and their families, revealing alterations in quality of life, physical and mental health, and social experiences. These include gastrointestinal symptoms, self-esteem issues, social concerns related to bullying and shame, anxiety, depression, and challenges in accessing healthcare.^7^

Some reviews have shown indicative rates of mortality and neurodevelopmental difficulties, predominantly in developed countries, with limited insights into broader aspects related to quality of life.^8,9^ Therefore, there is a need to delve deeper into understanding how these manifestations occur in a broader population, using them as a foundation and starting point for analyzing the lives and well-being of those affected, identifying convergences or divergences from the existing literature.

However, little progress has been made in recent decades, primarily due to the incompletely understood definition and etiology of the disease.^5^ Consequently, addressing this issue becomes a priority, especially concerning short- and long-term outcomes to establish prevention strategies, potential treatments, and mitigation of complications. In this context, this study aims to describe the repercussions on the quality of life of patients affected by NEC during the neonatal period.

## METHOD

This is an integrative review for which a guiding question was formulated and a literature search was conducted, followed by data collection, critical analysis of the included studies, discussion of the results, and presentation of the review.^10^ The guiding question was: “What are the repercussions on the quality of life of patients affected by NEC in the neonatal period?”

To this end, we searched for studies published from 2012 to 2022, aiming to encompass the recent period with more research on the topic, on the Regional Portal of the Virtual Health Library (BVS), in the Latin American and Caribbean Health Sciences Literature database (LILACS), as well as Medical Literature Analysis and Retrieval System Online (Medline) and PubMed, in order to increase the possibility of locating studies from developing countries such as Brazil. We used Health Sciences Descriptors (DeCS): “necrotizing enterocolitis,” “quality of life,” and “prognosis” combined with the Boolean operator AND and the operator OR, in the following configuration: “Necrotizing Enterocolitis” AND (“quality of life” OR “prognosis”).

We included articles that addressed the guiding question of our research: “What are the repercussions on the quality of life of patients affected by NEC in the neonatal period?”, with neonates as the population (P); being affected by NEC as the exposure €; repercussions on quality of life as the outcome (O); and observational studies as the study type (S), following the PECOS^11^ strategy. We considered articles published from 2012 to 2022 and available for full-text reading. We excluded duplicated articles, reviews, animal studies, studies unrelated to children or NEC, articles with outcomes other than quality of life, studies addressing the topic with comorbidities, and articles unavailable for complete reading.

This selection process was carried out by two independent and blinded evaluators who, after selection, resolved selection discrepancies according to inclusion criteria, beginning with the title and abstract, using the Rayyan^12^ platform as a tool. Subsequently, the selected articles were read in their entirety. Then, they were organized according to the following variables: author and year of publication, study design and location, sample size, and key findings, with the creation of a Microsoft Excel® spreadsheet for data compilation.

## RESULTS

**Figure 1:**
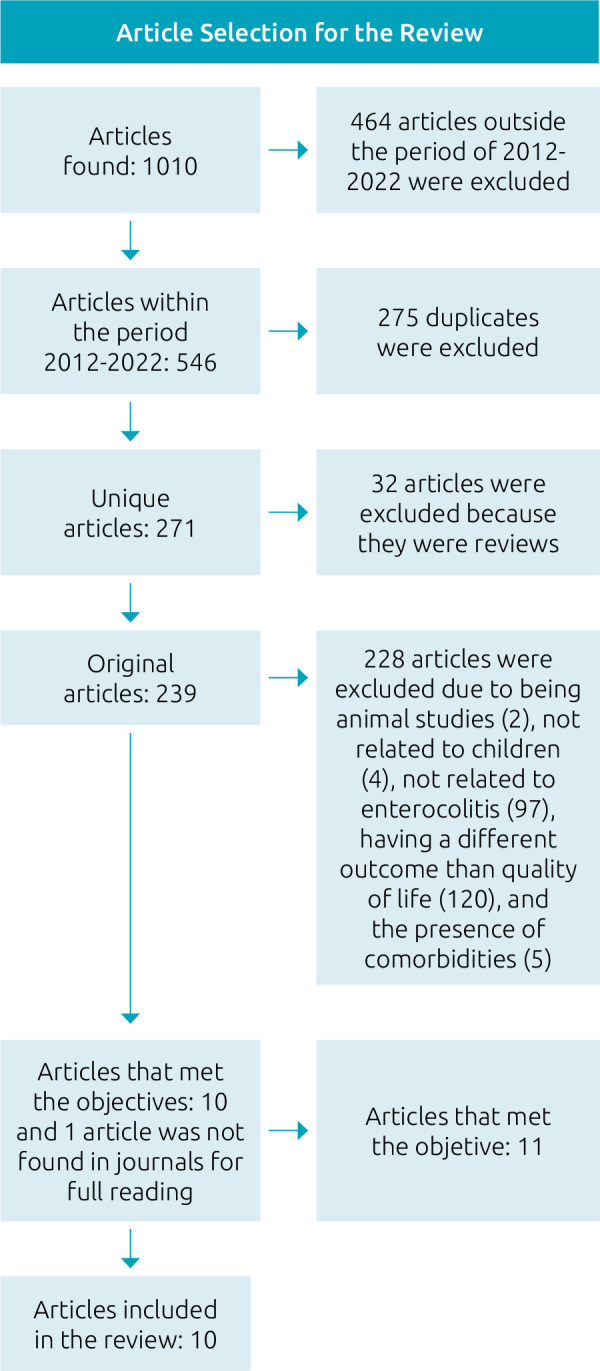
Study selection flowchart.

A total of 1,010 studies were identified, of which ten were selected to comprise the bibliographic sample for this review. Figure 1 illustrates the screening process.

The study sample includes six countries: the United States, the United Kingdom, Japan, Canada, China, and Germany. The studies were published between 2014 and 2021, but the follow-up for some of them began earlier, with examples of retrospective studies starting in 2002 and 2006. The sample in this work encompasses 767 children diagnosed with NEC. Among these studies, there is a predominance of the analysis of newborns,^13-15^ with some studies observing infants^16^ and others tracking their development during childhood,^17-19^ or even the beginning of adolescence.^20^ Table 1 and Table 2 present the individual characterization of the studies.

**Table 1:** Characterization and main conclusions of studies focused on survival and quality of life.

Authors	Study design and location	Sample	Main results and conclusions
Neam et al.^17^	Seattle, United States Prospective study	Families of 91 children > 6 years (26 with NEC).	The quality of life of children with intestinal failure is lower when compared to the healthy population. This reduction in quality of life was found to be greater in school-age children.
Muto et al.^13^	Japan Retrospective Chart Review	82 cases of low birth weight newborns who underwent laparotomy due to NEC, FIP, and MRI	Delays in the development of fine and gross motor functions were more noticeable when compared to other functions. Showing a tendency for postural-motor decline between 1.5 and 3 years
Han et al.^20^	Boston, United States Prognostic study	268 infants with surgical NEC.	There was higher survival in patients who were not placed on unspecified comfort measures but underwent multiple surgeries. Overall survival for severe surgical NEC was 68%. Of these, 32% achieved enteral autonomy without transplantation. Of surgery survivors, 71% did not have severe neurological impairment, but dependence on PN was associated with a higher number of seizures, medications, and ongoing neurological follow-up.
Amin et al.^21^	United States Prospective observational study	241 patients, of whom 23 developed NEC.	The study demonstrated a reduction in quality of life with an increase in the length of hospital stay. Those who underwent more than one surgical intervention had higher quality of life compared to those who only underwent drainage or a single-stage laparotomy. After the age of 12, quality of life is significantly impaired.
Sparks et al.^19^	Boston, United States Comparative study	109 patients enrolled in a multidisciplinary intestinal rehabilitation program, with short bowel syndrome, among whom 37 were diagnosed with NEC.	45 patients (41%) achieved enteral autonomy after a median duration of PN of 15.3 months. Of NEC patients, 64.9% achieved enteral autonomy compared to 29.2% of patients with a different primary diagnosis. NEC patients can achieve autonomy even after extended periods of parenteral support.

NEC: necrotizing enterocolitis; FIP: focal intestinal perforation; MRI: meconium-related ileus; PN: parenteral nutrition.

**Table 2:** Characterization and key findings of studies focused on neurodevelopment and cognition.

Authors	Study design and location	Sample	Main results and conclusions
Mondal et al.^16^	United Kingdom Retrospective review, cohort	67 infants with NEC. Of those, 46 were born alive, and 18 were contacted.	The overall mortality rate was 31%, and 61% had some form of neurological impairment. Cognitive function was the most commonly compromised, at 56%. Surgical management was a risk factor for lower quality of life. The primary areas of impairment included special educational needs, cerebral palsy, blindness, speech difficulties, and learning difficulties. This developmental delay can be attributed to prematurity.
Zozaya et al.^14^	Canada Retrospective cohort study	2,019 premature newborns (22 and 28 weeks). Of these, 61 had perforated NEC, and 115 had non-perforated NEC.	Neonates had a higher rate of dysplasia severe bronchopulmonary disease, severe retinopathy of prematurity and infections. When compared to neonates without NEC, neonates with NEC had a smaller head circumference and lower scores for motor, cognitive and language development.
Lee et al.^15^	United States Secondary retrospective analysis	155 premature infants with prenatal or neonatal infections, of whom 10 (6.5%) had NEC, and 7 (4.5%) had NEC with sepsis.	Both groups were associated with longer time on parenteral nutrition. Patients with sepsis required longer ventilation time. Neonatal infections — including NEC — are associated with structural changes in the brain, but not with neurobehavioral changes.
Lin et al.^18^	China Longitudinal study	83 premature infants with NEC who survived. They were enrolled and divided into a surgical group (n=57) and a non-surgical group (n=26).	Of the infants, 37% were underweight, and the rate of weight loss in the surgical group was higher than in the non-surgical group; 27% were delayed in length and 17% lagged in head circumference; 22% had motor delay/ developmental disabilities, and the incidence rate in the surgical group was higher than in the non-surgical group (28 vs. 8%). Also, 6% were diagnosed with cerebral palsy, mostly in the surgical group.
Allendorf et al.^22^	Germany Retrospective case-control study	74 participants, consisting of 37 patients with NEC. 13 in group A (NEC treated conservatively), and 24 in group B (surgically treated NEC). 37 in the control group.	Patients in group A had higher rates of psychomotor and mental development compared to group B. Healthy patients had higher rates of mental development than both groups. Group B had a longer period of parenteral nutrition and risk of cognitive delay.

NEC: necrotizing enterocolitis

### Mortality, morbidity, and quality of life

Some studies relate enterocolitis to quality of life, indicating its detrimental impact on the lives of these children.^20,21^ In a prospective study conducted in the United States, it was concluded that children with intestinal failure (with enterocolitis being the second leading cause) experience a reduced quality of life when compared to healthy children. This reduction is linked to medical procedures, primarily during the school years, further widening the disparities between healthy children and those undergoing treatment.^17^ Regarding mortality, numerous studies have highlighted the high mortality rate of enterocolitis, with rates of 31,^16^ 32,^13,14^ and nearly 20% in another review,^20^ underscoring the severity of this condition. In terms of morbidity, some studies have reported a higher incidence of certain diseases in neonates with enterocolitis, such as bronchopulmonary dysplasia, severe retinopathy of prematurity, and infection.^13,14^ Enterocolitis has also been associated with post-surgical low weight and shorter length, as well as cases of cerebral palsy.^18^ In another study, a comparison was made between comfort measures and surgical intervention as therapeutic approaches, with surgery demonstrating higher survival rates.^20^ In the same study, the achievement of total parenteral nutrition autonomy without intestinal transplantation was observed, and it was concluded that dependence on total parenteral nutrition was linked to a higher number of seizures, medications, and the need for more continuous neurological follow-up.^20^ In line with this, another cohort study analyzed enterocolitis in conjunction with other pathologies, and it showed a greater likelihood of enteral autonomy, particularly when the diagnosis of enterocolitis is associated with SBS.^19^

### Physical development

Regarding motor development, children with NEC have delayed development compared to healthy children.^13,14,18,22^ This development is even more delayed and has more deficiencies in groups that undergo surgery when compared to non-operated groups.^18,22^ In another study, it was concluded that motor development is less affected in older children,^16^ which corresponds to a similar study in Japan that also found delays in fine and gross motor development, which were more noticeable compared to other functions.^13,14^

### Neurodevelopment

Several studies found results related to the neurodevelopment of children with NEC. It was identified that children with NEC experienced impairment in cognitive function, with those undergoing surgical management being more affected compared to those managed on an outpatient basis.^16,22^ The main compromised aspects included special educational needs, cerebral palsy, and blindness.^16^ A U.S. prognostic study reported that the majority (71% in this study) of surgery survivors did not have severe neurological impairment, but dependence on parenteral nutrition was associated with a higher number of seizures, medications, and continuous neurological follow-up.^20^ A neurodevelopmental assessment conducted in a Japanese retrospective study,^13^ using the Kyoto Scale of Psychological Development, showed stability in the cognitive-adaptive domain with some delays, and the language-social domain was above the threshold with promising expectations. A similar study conducted in Canada^14^ based on the Bayley III scale identified lower scores in motor, cognitive, and language development in children with NEC.

The located studies also neuroanatomically addressed the development of children with NEC, reporting a reduction in head circumference,^14,18^ a decrease in biparietal diameter and transcerebellar diameter, as well as an increase in the left ventricle.^15^

In most studies, the sample consists exclusively of preterm infants,^13-16,18^ these being less than 29 weeks’ gestational age, some with an average of 26 weeks, others 24 weeks. This can lead to ambiguity regarding the results of neurodevelopment, whether NEC exacerbates the already known deficits caused by prematurity, or if this compromise in neurodevelopment is heightened in preterm infants with NEC when compared to preterm infants only.

### Key findings

**Figure 2:**
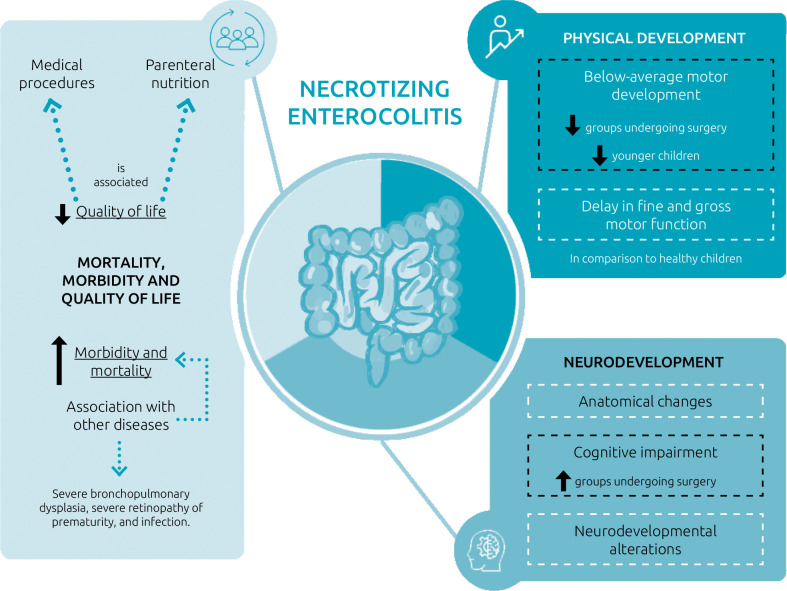
Primary long-term outcomes related to necrotizing enterocolitis, in addition to high mortality, include delays in neurodevelopment with cognitive, physical, and motor repercussions, impacts on the gastrointestinal tract, with the need for parenteral nutrition being the most frequently described.

Figure 2 describes the primary long-term outcomes in patients affected by NEC. Children with this disease are prone to experiencing cognitive impairment,^13,14,16,18,22^ with more significant findings observed in patients who underwent surgical procedures due to more severe conditions.^14,15,18^ Regarding motor development, it was also considered below average when compared to healthy children,^13,14,18^ with a more noticeable delay in the development of fine and gross motor functions.^13,14^ Thus, the search for the relationship between NEC and quality of life resulted in the finding that this condition has a negative impact on the well-being of affected individuals.^20,21^

## DISCUSSION

Upon the analysis of data related to NEC, it is evident that this condition contributes to high morbidity and mortality rates, with severe complications resulting from extensive intestinal necrosis, as well as undesirable long-term consequences that impact the quality of life of children. Studies report a high prevalence of NEC-related mortality, with approximately 30% mortality in the United Kingdom (31%), Japan (32%), and the United States (20%), indicating the gravity of this condition.^13,14,16,20^ A study conducted in the United States revealed that NEC is the second leading cause of reduced quality of life when compared to other intestinal pathologies. This allows for the identification of differences between healthy children and those with NEC who need to undergo treatments or medical procedures during the school years.^17,20,21^ Although NEC has been correlated as a condition detrimental to quality of life, few studies have addressed the multidimensional long-term impacts of NEC complications on patients’ lives. This highlights the need for research that addresses this aspect in a way that can assist in the development of strategies and approaches to improve the prognosis of these patients.

Given its severity and increasing mortality, it has been possible to analyze some diseases related to morbidity that are associated with NEC, such as bronchopulmonary dysplasia, severe retinopathy of prematurity, and infection. In addition, post-surgical low weight, shorter stature, and some cases of cerebral palsy have also been linked to NEC.^13,14,18^

In view of this, measures of comfort have been analyzed in relation to the surgical approach, as well as the autonomy of parenteral nutrition without intestinal transplantation, since dependence was associated with a higher number of seizures, medications, and the need for more continuous neurological monitoring. One study addressed NEC in conjunction with other pathologies and found a greater propensity for enteral autonomy, particularly when associated with SBS.^19,20^

There is very limited data on the gastrointestinal sequelae of NEC, with the notable exception of SBS and/or intestinal failure (IF).^23^ The relationship between NEC and SBS deserves special attention. This syndrome is one of the major complications secondary to NEC, in which extensive resection was necessary, leading to segmental loss after resection and subsequent mucosal hyperplasia. Therefore, prolonged parenteral nutrition is often required due to the difficulty of intestinal readaptation to enteral nutrition, sometimes for indefinite periods.^24^ In these patients, the multidisciplinary contribution of a dedicated nutritional support team is an important aspect of care.^25^ A novel study^7^ conducted with survivors of NEC and parents of children affected by NEC from various countries identified digestive repercussions as the most relevant cause of a decline in patients’ quality of life, due to the need for long-term enteral or intravenous nutrition. In children with SBS, an interdisciplinary team becomes essential to provide a more comprehensive approach to the child, thereby allowing for the integration of a broader perspective to enhance the quality of life for the child and their family.^26^

The importance of breast milk for premature infants is emphasized, which is associated with a reduced risk of infections and the development of NEC, as well as providing appropriate nutrients for the baby.^27^ Colostrum therapy is also highlighted as an important measure in the prevention of NEC, and mothers should be encouraged to provide it for the child’s protection.^28^

Regarding neurological development, it is demonstrated that children with NEC have impaired development compared to healthy children,^13,14,18,22^ with more visible and pronounced deficits, including more disabilities and developmental delays, in children who have undergone surgery than those who have not.^18,22^ A systematic review^9^ analyzed five studies with results on neurodevelopmental deficits in NEC patients, with deficits reported in four of these studies, ranging from 24.8 to 61.1% of a total of 1,370 patients. There are discrepancies concerning issues related to motor development impacts, as some studies suggest lesser consequences in older children, while another study found greater delays in fine and gross motor development compared to other functions.^13,14,16^

Furthermore, research reveals that children with NEC exhibited impairment in cognitive function, with those undergoing surgery being more affected than those who did not.^16,22^ A study^29^ conducted in Brazil using the Bayley III scale to assess the cognitive development of premature patients aged six to 12 months described that the occurrence of NEC was inversely associated with cognitive performance in preterm infants at 12 months. Concerning neurological impacts, special considerations include individualized special needs, cerebral palsy, and blindness.^16,18^ Despite surgery not causing as much neurological impairment for survivors, parenteral nutritional dependence was associated with a higher incidence of seizures, medication use, and continuous neurological monitoring.^20^

A retrospective study conducted in Japan, focusing on the assessment of neurodevelopment using the Kyoto Psychological Development Scale, revealed specific domains analyzed, such as cognitive-adaptive, which demonstrated stable growth with some delays, and social language, with promising expectations. The study involved 82 cases of low birth weight, premature neonates with an average gestational age of 24,5 weeks. In a similar study in Canada, which examined 2,019 premature neonates with gestational ages between 22 and 28 weeks, an evaluation based on the Bayley-III scale identified impairments in cognitive, language, and motor development aspects in children with NEC.^13,14^ A multicenter study conducted in the United States with extremely low birth weight preterm infants indicated a relationship between NEC and developmental delays, particularly associated with alterations in the neurological physical examination and low scores on the Bayley scale, possibly due to vasoconstriction and hypoxic-ischemic events caused by intense inflammatory reactions.^30^

Additionally, there is an association between developmental deficits and anatomical issues in children with NEC, such as a reduced head circumference, decreased biparietal and transcerebellar diameters, as well as an increase in the left ventricle.^14,15,18^ These findings underscore the importance of early diagnosis to reduce disease progression and achieve a better prognosis.

In summary, it is evident that children with NEC have a higher tendency to exhibit cognitive impairment, with more pronounced outcomes in patients who have undergone surgical procedures due to more severe cases, accompanied by neuroanatomical findings that correspond to the deterioration of the condition.^13,16,18,22^ Additionally, motor development was considered below average compared to healthy children, with more noticeable delays in fine and gross motor functions. Although prematurity has a significant impact on outcome analysis, NEC has a detrimental effect on the developing brain, resulting in worse neurological development outcomes compared to preterm babies who do not develop NEC.^9^

The biopsychosocial aspects were not as well addressed in the analyzed studies. In the group of patients from the North American study^17^ involving 26 children diagnosed with NEC, quality of life was reduced, especially in those of school age, due to the need for absences for medical follow-ups and procedures, as well as aspects related to the use of feeding tubes and gastrostomy bags. Another study^21^ conducted in the United States with 23 patients affected by NEC pointed to this decline in the quality of life after the age of 12. The research^7^ conducted with survivors of NEC and parents of children affected by NEC from around the world described the negative impact on the quality of life of these patients related to the constant need for hospitalizations and lifelong dependence on parenteral nutrition. In this study, 80% of the interviewed survivors reported anxiety or body image concerns, and for 74% of a total of 143 parents, the physical or mental health was affected by the long-term complications in the lives of their children affected by NEC.

Given the complexity with which NEC impacts the lives of survivor patients, the lack of studies in this area is evident. In addition to the high mortality rate, survivors contend with repercussions that extend not only to the gastrointestinal tract with scarring and dependence on parenteral nutrition but also with impairment in neurological development, resulting in physical and motor repercussions, leading to various hospitalizations and impacting the social life of the patient and their family.

This issue also highlights the scarcity of studies addressing the importance of a multidisciplinary team in lifelong patient care, underscoring the need for the development of new studies that emphasize the importance of a patient-centered approach in identifying later complications and early intervention and support for these patients.^31^ This includes the benefits of a standardized and multidisciplinary approach to infants, encompassing individualized nutritional management, developmental assessment, and necessary developmental delay interventions.^23^

Being a review, limitations of the included studies may affect the results of this research. It is important to note that there is a restriction in the examined studies, with a lack of focus on psychosocial aspects and the consequences of parenteral nutrition, particularly in short bowel syndrome. Another limitation is that some studies^13,14,17,18,21,22^ also examined other diseases, thus not exclusively addressing patients with NEC. It is worth noting as another significant bias that both premature and full-term babies were included in the studies,^16^ which can influence the outcomes related to developmental changes. Therefore, further research is needed to fill these gaps and provide a more comprehensive understanding of the impact of NEC on children’s quality of life.

In conclusion, NEC has proven to be a severe condition contributing to high rates of morbidity and mortality in newborns and can lead to serious complications that impact the quality of life of affected children. These consequences are more closely associated with premature and low-birth-weight newborns and can cause a range of interferences, from physical to neurodevelopmental.

It has been evidenced that children with NEC exhibit below-average cognitive and motor development, with more pronounced delays in patients requiring surgery. Additionally, neurological impairment, special needs, cerebral palsy, and compromised cognitive function were also observed in children with NEC. Furthermore, the dependence on total parenteral nutrition is also highlighted as one of the main causes of a reduction in the quality of life of patients. This underscores the impacts that the pathology has on the quality of life of survivors, highlighting the need for further studies to develop improved therapeutic support strategies and to assess the lifelong development of these patients subject to the repercussions associated with NEC.
